# Co-Administration of Mavacamten and Tacrolimus in a Renal Transplant Recipient With Obstructive Hypertrophic Cardiomyopathy

**DOI:** 10.1016/j.jaccas.2026.110345

**Published:** 2026-10-09

**Authors:** Andrew Willeford, Arman Faravardeh, Jorge Silva Enciso, Quan M. Bui

**Affiliations:** a UC San Diego Skaggs School of Pharmacy and Pharmaceutical Sciences, La Jolla, California, USA; b SHARP Kidney Transplant Center, San Diego, California, USA; c Division of Cardiovascular Medicine, Department of Medicine, UC San Diego Health, San Diego, California, USA

**Keywords:** cardiomyopathy, echocardiography, genetic disorders, treatment

## Abstract

**Background:**

Mavacamten is a cytochrome P450 (CYP)3A4 inducer with a product label recommending careful monitoring alongside CYP3A4 substrates. Data characterizing its interaction with tacrolimus, a narrow-therapeutic-index immunosuppressant and CYP3A4 substrate, are lacking.

**Case Summary:**

A 62-year-old woman kidney transplant recipient with symptomatic obstructive hypertrophic cardiomyopathy was started on mavacamten 5 mg daily. Over 5 months, serial monitoring showed stable, therapeutic tacrolimus troughs (5.9-7.8 ng/mL) without dosage adjustments, alongside significant clinical improvement in obstructive symptoms.

**Discussion:**

This case uniquely details the longitudinal pharmacokinetic interaction between mavacamten and tacrolimus. This is highly relevant because pharmacokinetic models suggest CYP3A4 induction is dose-dependent; thus, safety demonstrated at 5 mg may not apply to higher regimens like 15 mg.

**Take-Home Messages:**

Low-dose mavacamten and tacrolimus coadministration is feasible. However, rigorous tacrolimus therapeutic drug monitoring remains essential, especially during mavacamten dose escalation.

## Visual Summary

**Figure:**
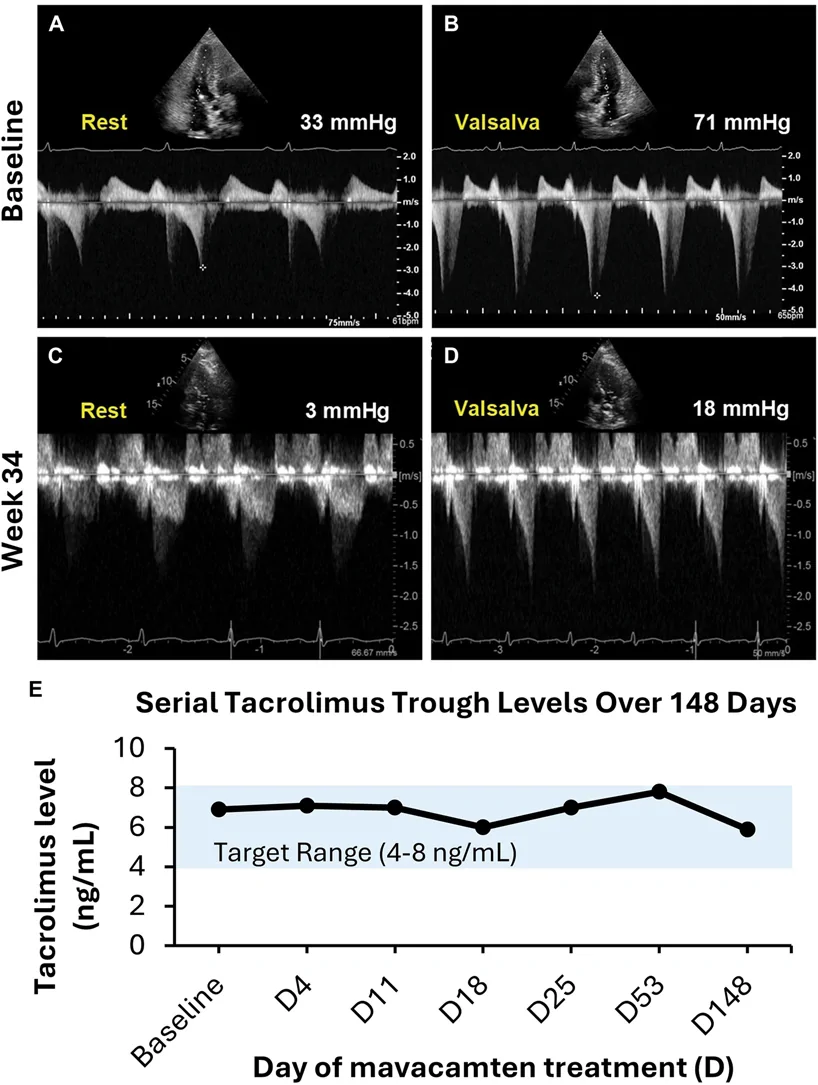

## History of Presentation

A 62-year-old woman presented to the cardiology clinic with symptoms of fatigue and shortness of breath on exertion despite medical therapy with metoprolol succinate 50 mg daily for her obstructive hypertrophic cardiomyopathy (HCM). She denied any chest pain or shortness of breath at rest. Vital signs included a blood pressure of 135/59 mm Hg and heart rate of 63 beats/min. Cardiovascular exam revealed a regular rate and rhythm with a grade 2 of 6 systolic murmur. Her lungs were clear to auscultation, and she had no jugular venous distension or peripheral edema.

## Past Medical History

The patient had a history of obstructive HCM diagnosed in 2024, as well as end-stage renal disease secondary to type 2 diabetes, status post deceased donor kidney transplant in 2021. Her maintenance immunosuppression regimen included tacrolimus 3 mg twice daily, mycophenolic acid 180 mg twice daily, and prednisone 5 mg daily. Her cardiac history was further complicated by sick sinus syndrome, status post permanent pacemaker implantation, alongside hypertension and hyperlipidemia. There was no known family history of HCM, and prior genetic testing did not reveal any clinically significant variants.

## Differential Diagnosis

Given the patient's presentation of worsening dyspnea and fatigue, the differential diagnosis primarily focused on the progression of her dynamic obstruction or worsening diastolic dysfunction related to her known obstructive HCM. Other considerations included uncontrolled hypertension, potential allograft dysfunction or inadequate immunosuppression leading to subclinical renal rejection, and pacemaker malfunction. Tacrolimus-induced left ventricular hypertrophy has been described in the literature and was also considered a cause of her worsening symptoms.^1^

## Investigations

The patient had variable clinic and home blood pressure readings but was mostly at goal below 130/80 mm Hg on metoprolol. Her renal function was stable, and tacrolimus trough levels were consistently at goal between 4 and 8 ng/mL for over a year leading up to presentation, arguing against subclinical allograft rejection or acute kidney injury as a contributor to her symptoms. Her dual-chamber pacemaker, as evaluated by her electrophysiologist the month before, was appropriately pacing and sensing. Diagnostic workup also included a baseline echocardiogram before presentation while on metoprolol, which revealed a left ventricular ejection fraction of 71%, a resting midcavitary gradient of 33 mm Hg, and a Valsalva gradient of 71 mm Hg. Cardiac magnetic resonance imaging demonstrated a maximum left ventricular wall thickness of 15 mm in the basal to midinferoseptal wall. Additionally, diffuse, patchy midmyocardial late gadolinium enhancement was present, qualitatively estimated to involve >15% of the total myocardial mass. Ultimately, her symptoms were thought secondary to progression of her obstructive HCM.

## Management

Due to her refractory symptoms and the inability to tolerate higher doses of beta-blockers, the patient was initiated on mavacamten 5 mg daily. Because mavacamten can induce cytochrome P450 (CYP) 3A4, there was a strong clinical concern for increased tacrolimus metabolism, a CYP3A4 substrate.^2^^,^^3^ To closely monitor for this potential interaction, serial tacrolimus trough levels were obtained on days 4, 11, 18, 25, 53, and 148 after starting mavacamten. This extended period of monitoring was designed to account for the long half-life of mavacamten (6-9 days) and to ensure that any changes in tacrolimus plasma levels were captured as mavacamten approached steady-state concentration.^2^ Concurrently, standard safety monitoring was scheduled according to the mavacamten risk evaluation and mitigation strategy program, with echocardiograms set for weeks 4, 8, 12, and 34 following the initiation of therapy.

## Outcome and Follow-Up

The serial tacrolimus troughs remained therapeutic, ranging from 5.9 to 7.8 ng/mL (goal 4-8 ng/mL) without requiring any dose adjustments. All levels were drawn appropriately before the patient’s morning dose of tacrolimus, and the patient endorsed strict adherence, with no other interacting therapies added to her regimen. By week 8, the patient reported significantly improved energy and greater walking tolerance, and she denied further dyspnea. This symptomatic improvement was sustained through week 34, which correlated with an overall improvement in resting and Valsalva gradients from baseline to the time of writing this report (Figure 1). Her left ventricular ejection fraction remained stable and above 50% throughout the entire mavacamten treatment course (Figure 2). Continuous-wave Doppler echocardiogram images at week 34 are shown. The patient currently remains clinically stable on tacrolimus 3 mg twice daily and mavacamten 5 mg daily, and she will continue standard therapeutic drug monitoring (TDM) every 3 months, alongside risk evaluation and mitigation strategy-mandated echocardiography for each respective therapy.

**Figure 1:**
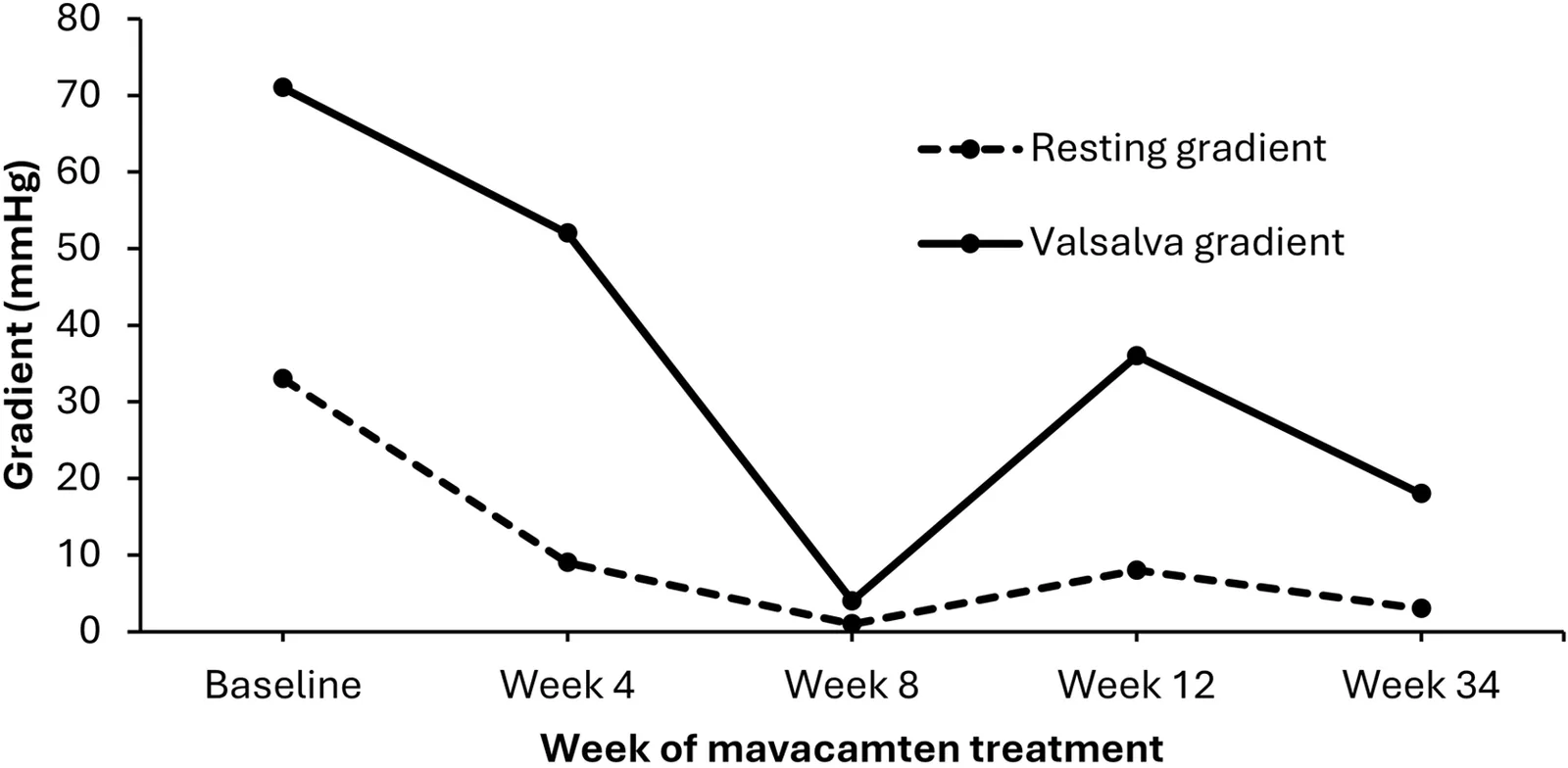
Resting and Valsalva Midcavitary Gradients While on Mavacamten Gradients were measured using transthoracic echocardiography in accordance with the mavacamten risk evaluation mitigation strategies program. Mavacamten was dosed at 5 mg daily throughout the timeline.

**Figure 2:**
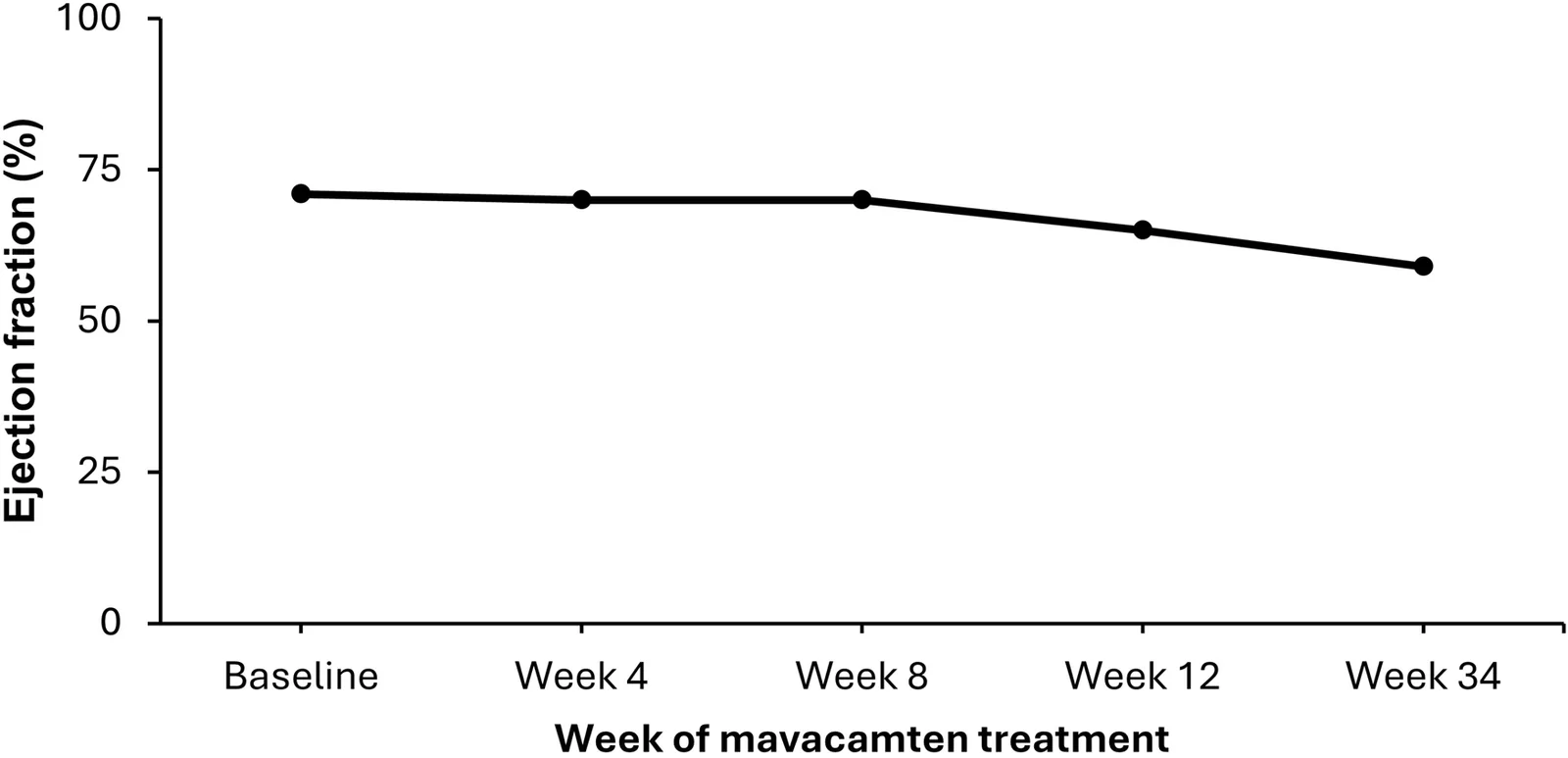
Left Ventricular Ejection Fraction While on Mavacamten Left ventricular ejection fraction was measured using transthoracic echocardiography in accordance with the mavacamten risk evaluation and mitigation strategies program and remained above 50% throughout treatment.

**Visual Summary:**
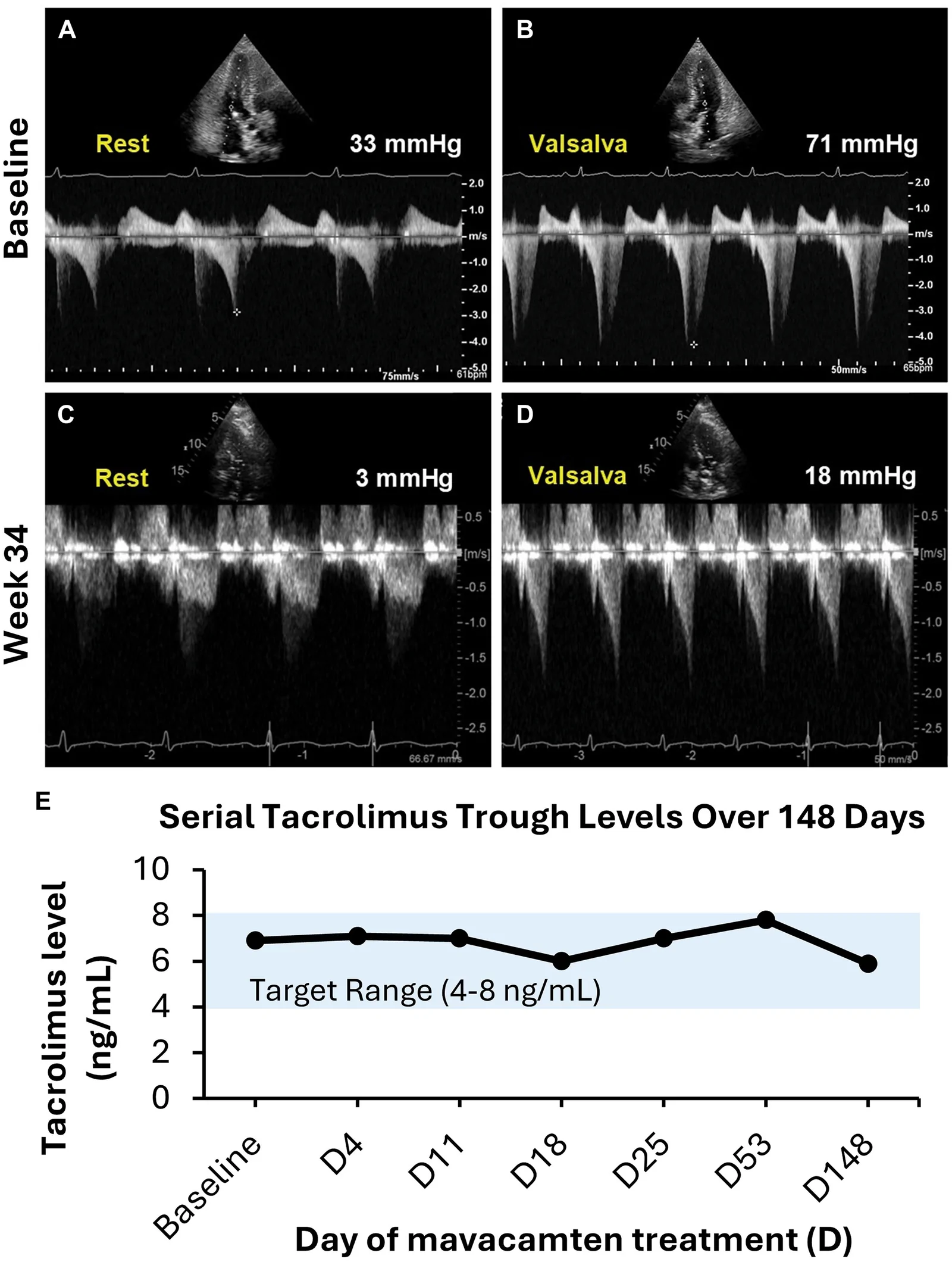
Timeline of Echocardiographic and Tacrolimus Trough Level Changes After Starting Mavacamten Continuous-wave Doppler through the midcavitary region at rest (A) and with Valsalva (B) before mavacamten treatment. Midcavitary gradients at rest (C) and with Valsalva (D) after 34 weeks of mavacamten treatment. Tacrolimus trough levels (E) were drawn after initiation of 5 mg daily mavacamten and remained between 5.9 and 7.8 ng/mL without dose adjustment. Mavacamten was dosed at 5 mg daily throughout the timeline.

## Discussion

This case describes the successful management of a 62-year-old woman deceased donor kidney transplant recipient with symptomatic obstructive HCM treated with mavacamten while on a tacrolimus-based regimen. While mavacamten use has been noted in an orthotopic heart transplant patient, that report described a patient maintained on sirolimus and lacked details regarding tacrolimus TDM.^4^ To our knowledge, this is the first case to specifically describe the longitudinal pharmacokinetic profile of tacrolimus following mavacamten initiation. Despite mavacamten’s role as a CYP3A4 inducer, serial monitoring over 5 months showed stable tacrolimus troughs (5.9-7.8 ng/mL) without dose adjustments.

This stable profile is notable given the metabolic pathways of both agents. Tacrolimus is a narrow therapeutic index immunosuppressant that is highly susceptible to drug-drug interactions via the CYP3A4 pathway, such that a CYP3A4 inducer typically accelerates its clearance and risks graft rejection.^5^ Mavacamten, which is primarily metabolized via CYP 2C19 (74%), 3A4 (18%), and 2C9 (8%), is itself vulnerable in this way.^2^ The label contraindicates strong CYP 2C19 inhibitors and moderate-to-strong 2C19 or 3A4 inducers and requires mavacamten dose reduction when initiating a weak-to-moderate 2C19 inhibitor or a moderate-to-strong 3A4 inhibitor. Mavacamten also acts as a mild-to-moderate inducer of these same enzymes, but the labeling offers only a general recommendation without agent-specific guidance to closely monitor CYP3A4, 2C9, and 2C19 substrates.^2^ This case fills that gap by providing longitudinal, drug-specific evidence for tacrolimus coadministration.

The conservative labeling precaution to monitor for decreased effectiveness of CYP3A4, 2C9, and 2C19 substrates is driven by mavacamten’s unique pharmacokinetics in its target study group. Systemic mavacamten exposure is approximately 2.7-fold higher in patients with HCM than in healthy volunteers.^6^ While short-term, 16-day studies in healthy subjects showed minimal impact on CYP3A4 substrates,^7^ physiologically based pharmacokinetic modeling suggests that sustained, higher-dose therapeutic use (eg, 15 mg daily for 50 days) in patients with HCM leads to greater enzyme induction, resulting in an estimated 45% reduction in substrate exposure.^6^

The absence of a clinically significant interaction in our patient is likely attributable to the relatively low dose of mavacamten used (5 mg daily). In vitro data demonstrate that CYP3A4 induction occurs primarily at concentrations much higher than those achieved with standard low-dose therapy.^8^ This dose-dependent threshold is further supported by the physiologically based pharmacokinetic modeling, which predicts that higher mavacamten regimens (15 mg daily) are required to meaningfully decrease the area under the curve and maximum concentration of sensitive CYP3A4 substrates.^6^ Consequently, the 5 mg daily dose administered to our patient was likely insufficient to induce CYP3A4 to a degree that would alter tacrolimus clearance.

## Conclusions

While this case demonstrates that low-dose mavacamten safely provided significant symptomatic and hemodynamic relief without altering immunosuppressive coverage, it should not imply that monitoring can be relaxed. Because higher doses of mavacamten may cross the threshold for meaningful CYP3A4 induction, safety cannot be generalized across all dosing tiers or transplant recipients. Ultimately, this case supports the clinical feasibility of using mavacamten alongside background tacrolimus therapy, provided clinicians maintain rigorous TDM and serial trough monitoring, particularly during dose escalation.

## Funding Support and Author Disclosure

Dr Willeford serves as a consultant for Bristol Myers Squibb and Cytokinetics. Dr Faravardeh serves as a consultant for Veloxis. Dr Silva Enciso serves as a consultant for Bristol Myers Squibb. Dr Bui serves as a consultant for Bristol Myers Squibb, Papillon Therapeutics, and Myoventive.

**Take-Home Messages**

• At 5 mg daily, mavacamten does not reach the threshold required to induce cytochrome P450 3A4 metabolism, allowing tacrolimus troughs to remain stable without dose adjustments.
• Because higher mavacamten doses are predicted to significantly reduce tacrolimus levels, routine trough monitoring is recommended, especially when titrating to the higher end of the therapeutic range (eg, 15 mg).
